# Incidence and Survival of IDH-Wildtype Glioblastoma and IDH-Mutant Astrocytoma by Treatment and Sex: A Regional Study in Spain (2011–2021)

**DOI:** 10.3390/medsci13040233

**Published:** 2025-10-14

**Authors:** J. A. Encarnación, C. Manso, M. Royo-Villanova, P. Ruiz, M. I. De la Fuente, E. Cárdenas, S. Ros, J. L. Alonso-Romero

**Affiliations:** 1 Radiation Oncology Service, University Hospital Virgen Arrixaca, 30120 Murcia, Spain; isabeldelafuente@hotmail.com (M.I.D.l.F.); enrique.cardenas@carm.es (E.C.); 2 Biomedical Research Institute of Murcia, 30120 Murcia, Spain; clara92mm@hotmail.com (C.M.); mario.royo-villanova@carm.es (M.R.-V.); paula.ruiz@carm.es (P.R.); silverio.ros@carm.es (S.R.); josel.alonso2@carm.es (J.L.A.-R.); 3 Intensive Care Unit, University Hospital Virgen Arrixaca, 30120 Murcia, Spain; 4 Medical Oncology Service, University Hospital Virgen Arrixaca, 30120 Murcia, Spain

**Keywords:** glioma, glioblastoma, age-adjusted incidence, treatment-based mortality, survival

## Abstract

Background: The incidence and prognosis of high-grade gliomas differ according to histopathological and molecular features. The WHO 2021 CNS classification emphasized IDH status, but historical cohorts often lacked systematic molecular profiling. Methods: We conducted a retrospective population-based study including adult patients diagnosed with IDH-wildtype glioblastoma or IDH-mutant astrocytoma in a Spanish tertiary center (2011–2021). Incidence trends and survival outcomes were analyzed according to treatment modality and sex. Results: A total of 1057 patients were included: 530 (50.1%) with IDH-wildtype glioblastoma and 137 (13%) with IDH-mutant astrocytoma. Incidence of both subtypes significantly increased during the study period (*p* < 0.01). Median overall survival (OS) was 12.3 months for IDH-wildtype glioblastoma and 38.4 months for IDH-mutant astrocytoma. Multimodal therapy (surgery, radiotherapy, chemotherapy) significantly improved OS and progression-free survival (PFS) in both subgroups (*p* < 0.001). Male sex was associated with longer OS in both tumor types (*p* < 0.05). Conclusions: IDH-wildtype glioblastoma shows persistently poor outcomes despite increasing incidence, while IDH-mutant astrocytoma demonstrates better survival, particularly in male patients and those receiving multimodal therapy. These findings reflect real-world practice and provide epidemiological and survival data from Southern Europe to guide future clinical and public health strategies.

## 1. Introduction

Primary tumours of the central nervous system (CNS) represent a heterogeneous group of neoplasms with diverse histopathological and molecular features [1]. These tumours may affect both pediatric and adult populations, though their incidence, biological behavior, and prognosis differ significantly between age groups. In pediatric patients (aged 0–19 years) [2], CNS tumours constitute the most common solid malignancy and the leading cause of cancer-related mortality, with an incidence of 6.1 per 100,000 children and a balanced sex ratio [3]. In adults, malignant gliomas predominate, especially among individuals over 40 years of age, and tend to have more aggressive clinical behavior and poorer outcomes.

In the adult population, malignant gliomas account for approximately 60% of primary brain tumours. The most clinically relevant high-grade gliomas are glioblastoma (GB), classified as WHO grade 4, and astrocytomas with IDH mutation, which may be grade 3 or 4 depending on histologic and molecular criteria [4]. The recent 2021 WHO Classification of CNS Tumours places strong emphasis on molecular markers for the accurate diagnosis of diffuse gliomas, with IDH mutation status representing a central criterion [5]. Notably, the entity historically termed ‘anaplastic astrocytoma’ (AA) no longer exists as a distinct diagnosis in the current WHO classification. Tumours previously diagnosed as IDH-wildtype astrocytoma may now be reclassified as IDH-wildtype glioblastoma or assigned to other categories, depending on additional histopathological and molecular features. In contrast, IDH-mutant tumours are classified as ‘IDH-mutant astrocytoma’, with grading determined by an integrated assessment of both histologic and molecular criteria. Accordingly, any diagnosis of AA without molecular characterization is considered incomplete and does not conform to current diagnostic standards. Despite these advances, molecular characterization of diffuse gliomas has not been systematically performed in routine practice across all centers until recent years. In Spain, molecular testing for markers such as IDH1 R132H by immunohistochemistry (IHC) or sequencing, TERT promoter mutation, MGMT methylation, p53, ATRX, and 1p/19q co-deletion became progressively more available only after 2016 [6]. Therefore, studies involving earlier diagnostic periods must acknowledge the limitations in molecular classification and the use of “not otherwise specified” (NOS) designations, such as “GB, NOS” or “AA, NOS”, to reflect real-world historical diagnostic criteria. This study included patients diagnosed between 2011 and 2021, during which the application of WHO 2021 was not yet standard. While the absence of uniform molecular profiling prevents strict reclassification, the histopathological diagnoses were based on accepted criteria at the time of diagnosis and allow meaningful epidemiological and survival analysis based on clinical practice.

Epidemiologically, GB remains the most frequent and aggressive primary malignant brain tumour in adults, typically arising de novo and peaking in incidence around the sixth decade of life. Its annual incidence is estimated at 3–5 per 100,000 individuals, whereas IDH-mutant astrocytomas (previously categorized in part as AA) are less common, at approximately 0.5 per 100,000. Five-year overall survival (OS) rates remain poor: ~5.6% for GB and ~30% for IDH-mutant astrocytomas. Despite advances in surgical resection, radiotherapy (RT), and chemotherapy (CT), survival improvements over the past decades have been modest. Favorable prognostic factors include younger age, good performance status, lower histological grade, cerebellar location, and gross total resection [7].

Sex-based differences in incidence and survival outcomes have also been reported. Malignant gliomas show a slight male predominance, while women exhibit a higher overall frequency of primary brain tumours due to the predominance of benign tumours such as meningiomas [8]. The underlying mechanisms of these sex differences remain unclear but are under active investigation [3,8].

Recent epidemiological trends suggest an increase in the incidence of high-grade gliomas globally. In Spain, CNS tumour incidence has risen by approximately 12% since 2017 [4,8,9,10]. This increase may be partially explained by improved neuroimaging and diagnostic methods, but environmental, occupational, and demographic factors may also contribute [11,12]. CNS tumours represent approximately 2% of all cancers in adults in Spain and cause an estimated 3200 deaths annually [13,14,15,16,17].

Given this context, the present study aimed to examine the incidence and survival outcomes of histologically diagnosed IDH-wildtype glioblastoma and astrocytomas with IDH mutation between 2011 and 2021 in a Spanish adult population, using data from a regional healthcare registry covering 1.5 million inhabitants. The study included only patients aged 18 years or older and assessed survival in relation to sex and treatment modality. Although molecular classification was not uniformly available for the entire cohort, we acknowledge this as a limitation and provide detailed justification for the diagnostic criteria employed. This population-based analysis offers relevant insights into the historical epidemiology and real-world management of high-grade gliomas in Southern Europe.

## 2. Methods

### 2.1. Ethical Considerations

According to Spanish law, individual-level data with identifiers can only be used for scientific research with the approval of the authorities or for statistics. The data processing procedures were evaluated and approved by our hospital’s ethics committee before starting our study.

### 2.2. Patient Selection

We retrospectively reviewed the electronic medical records of all patients diagnosed with brain tumours with a histopathological diagnosis of IDH-wildtype glioblastoma or IDH-mutant astrocytoma at a tertiary care center in Murcia, Spain, between January 2011 and December 2020. Patients were followed until December 2023. For all included cases of diffuse gliomas diagnosed as anaplastic astrocytoma or glioblastoma, the IDH1 mutation status was retrospectively determined. Only tumors confirmed to harbor an IDH mutation (either by IHC or molecular techniques) were classified as IDH-mutant astrocytoma, in accordance with the 2021 WHO Classification of CNS Tumors. Cases lacking IDH mutations were categorized as IDH-wildtype glioblastoma if additional diagnostic criteria were met (TERT promoter mutation, EGFR amplification, or combined +7/−10 chromosomal alterations) [5].

Based on these findings, tumors were classified according to the 2021 WHO Classification of Tumors of the Central Nervous System. All IDH-mutant diffuse astrocytic tumors were reclassified under the umbrella term “IDH-mutant astrocytoma”, which includes WHO Grade 2, Grade 3, and Grade 4 tumors.

For patients diagnosed between 2011 and 2016, when molecular testing was not yet uniformly available in our institution, IDH status was retrospectively determined whenever archival tissue was available. In this subgroup, archival tumor samples were successfully retrieved in 82% of cases. Patients for whom no archival material was available, or in whom molecular testing could not be completed (18%), were excluded from the study. As a result, all patients included in the final cohort had confirmed IDH status in accordance with the 2021 WHO CNS classification.

Patients with recurrent disease initially diagnosed before January 2011 and those lost to follow-up were excluded from the final analysis.

### 2.3. Clinical and Demographic Characteristics

Clinical and demographic data were collected from the electronic medical records, including: age at diagnosis, tumour histology, sex, date of diagnosis, date of progression, date of death, treatment received, extent-of-disease evaluation prior to treatment initiation, presence of cardiovascular risk factors (CVRF), active alcohol consumption, active smoking, diagnosis of secondary neoplasms, cause of death, and preoperative Eastern Cooperative Oncology Group (ECOG) performance status.

Overall survival (OS) was defined as time from diagnosis to death from any cause. Progression-free survival (PFS) was defined as time from diagnosis to radiological progression or death, whichever occurred first. 

### 2.4. Survival and Follow-Up

Patients were followed from the date of histopathological diagnosis until death, emigration out of the healthcare system, or until the end of 2023. Due to mandatory registries incorporating unique personal identifiers, complete follow-up data were available for the entire cohort.

### 2.5. Statistical Analysis

We calculated the standardised incidence of IDH-wildtype glioblastoma and astrocytomas with IDH mutation. Incidence rates were also reported by age groups (18–30, 30–40, 40–60, and >60 years). For all statistical analyses, a result was considered significant when *p* < 0.05. Differences in overall survival (OS) and progression-free survival (PFS) between groups were assessed using the log-rank test and represented with Kaplan–Meier curves. Subgroup analyses were performed for IDH-wildtype glioblastoma and IDH-mutant astrocytoma patients separately using univariate Cox proportional hazards models to evaluate associations between clinical variables and OS/PFS. Specific subgroup analyses by sex and treatment type were also conducted. Average survival times were reported. Differences in excess mortality between the two time periods were evaluated using the likelihood ratio test, with *p*-values adjusted for multiple comparisons using the Benjamini–Hochberg procedure. All analyses were conducted using SPSS statistical software (IBM SPSS Statistics for Windows, version 28.0).

## 3. Results

A total of 1784 patient records were initially identified. After reviewing the registry, 727 patients were excluded: 523 due to the presence of brain metastases from a primary extracranial tumour, and 205 because they lacked a pathological anatomy (PA) study, having been diagnosed solely by imaging. After these exclusions, 1057 patients were eligible for analysis. The distribution of final diagnoses is shown in Table S1 (In Supplementary Material). Among these, 530 patients (50.1%) were diagnosed with IDH-wildtype glioblastoma and 137 (13%) with IDH-mutant astrocytoma, comprising the two main high-grade glioma subtypes evaluated in this study. The remaining diagnoses included meningiomas, low-grade gliomas, and other primary CNS tumours.

### 3.1. IDH-Wildtype Glioblastoma Results

#### 3.1.1. Clinical and Demographic Characteristics

The median age at diagnosis of the sample was 68.22 years (range: 18–90, standard deviation (SD): 19.29). The median age of death was 62.35 years (range: 19–91, SD: 18.61). If we stratify IDH-wildtype glioblastoma into age groups of 18–30, 30–40, 40–60, and >60 years, we observe that patients over 60 years of age constituted the largest IDH-wildtype glioblastoma population (Table 1).

If we examine IDH-wildtype glioblastoma diagnoses per year to assess whether there has been an increase in incidence in recent years, we observe the following results: 2011: 44, 2012: 47, 2013: 43, 2014: 53, 2015: 52, 2016: 50, 2017: 52, 2018: 60, 2019: 62 and 2020: 67.

**Figure 1:**
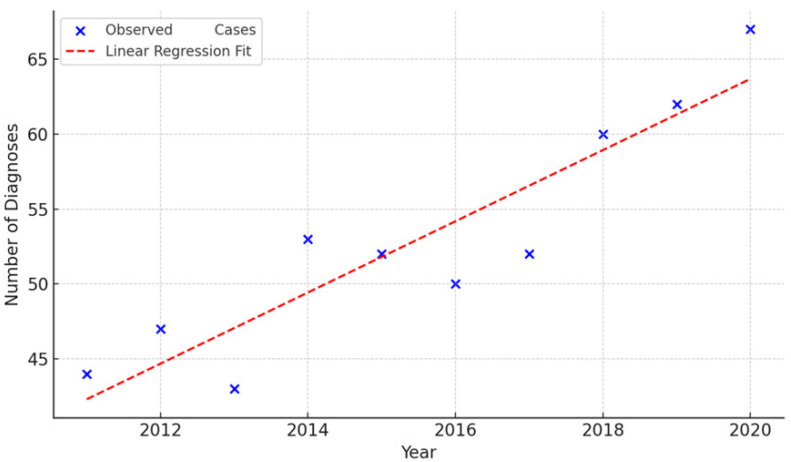
Annual IDH-wildtype glioblastoma Diagnoses (2011–2020).

To assess the trend in IDH-wildtype glioblastoma incidence over time, we performed a simple linear regression analysis using annual case counts from 2011 to 2020. The model showed a statistically significant upward trend, with an estimated increase of 2.38 cases per year (R^2^ = 0.84, *p* = 0.0002). This suggests a robust increase in IDH-wildtype glioblastoma diagnoses over the study period. Figure 1 displays the observed values and fitted regression line.

In our cohort, there was a slightly higher percentage of men than women (55.7% vs. 44.3%, respectively). Regarding patients’ functional status measured by the ECOG scale at diagnosis, 138 patients (26%) had an ECOG of 0, 322 (60.8%) had an ECOG of 1, and 70 (13.2%) had an ECOG of 2 or higher.

Fifty-eight patients (7.4%) had other primary neoplasms in addition to the primary CNS tumour, all of which were metachronous tumours. Of these 58 patients, 42 (6.3%) had a disease-free period greater than five years since the other tumour was diagnosed. None of the patients had metastatic disease of the primary non-CNS tumour.

Regarding the treatment received by patients, it is noteworthy that more than half of the patients with IDH-wildtype glioblastoma were treated with surgery + chemotherapy (CT) + radiotherapy (RT) (58%). The second most common option was incomplete surgery/biopsy without any further treatment. Concerning the specific oncological treatment received by each patient, we highlight that only 8.68% did not receive any type of CT and that more than half of the patients received 60 Gy of brain RT treatment. The percentage of patients per treatment is specified in Table 2.

Focusing on the type of CT received by patients, 29.8% were treated exclusively with temozolomide; 16.5% were treated with temozolomide + bevacizumab (bevacizumab in disease progression); 4.8% had bitherapy with temozolomide + fotemustine, administered at disease progression; and triple therapy (temozolomide + bevacizumab + fotemustine) was used in 10.1% of patients as sequential treatment in each progression. In total, 61.17% received treatment with temozolomide, either as monotherapy or in combination with another drug.

Regarding RT treatment, the most used fractionation in our series was 60 Gy (2 Gy fractions), which was administered to 339 patients (50.8%), of which 65 (12.26%) received a second RT treatment on the previously treated area. Two hundred twenty-one patients (33.2%) did not receive RT treatment during their oncological process. A total of 38 patients (7.16%) underwent a second surgical intervention.

Regarding the extension studies performed on patients, 448 patients (84.5%) underwent extension studies with Computed Tomography of the Thorax, Abdomen, and Pelvis (CT TAP) or Positron Emission Tomography–Computed Tomography (PET CT); none of these patients presented any sign of dissemination of the primary brain tumour outside the CNS. During follow up of the oncological disease, complementary studies were performed to evaluate any possible dissemination of the primary disease; none of these studies showed disease outside the CNS. Neuroaxis dissemination was observed in 13 patients (2.45%) via MRI following changes in their neurological symptoms.

As for the cause of death, 96.6% of cases were due to disease progression at the CNS level, representing 512 of the 530 IDH-wildtype glioblastoma patients. Mortality due to infections was the second cause in these patients, accounting for a total of 12 deaths (2.26%).

#### 3.1.2. Progression-Free Survival (PFS)

**Figure 2:**
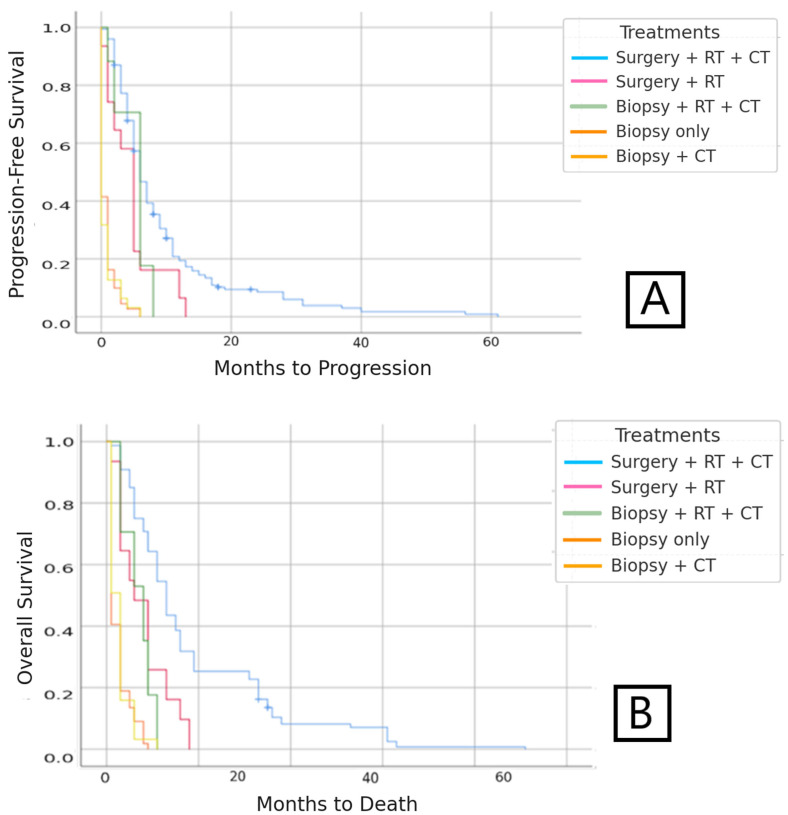
(**A**) PFS according to type of treatment in patients with IDH-wildtype glioblastoma. (**B**) OS according to type of treatment in patients with IDH-wildtype glioblastoma.

Median PFS was 6.26 months (95%CI: 5.7–7.8). The univariate analysis showed that patients who received treatment with complete surgery + RT + CT had the longest relapse-free period, with a median of nine months. A statistically significant worst prognosis of <1 month was found in patients treated with biopsy +/− CT (*p* < 0.05) (Figure 2).

Regarding sex, no significant differences were found in terms of mean PFS (*p* = 0.458), being 5.9 months in women and 6.4 months in men.

#### 3.1.3. Overall Survival (OS)

Median OS was 12.3 months (95%CI: 11.3–13.8) in patients diagnosed with IDH-wildtype glioblastoma. The stratified study conducted on these IDH-wildtype glioblastoma patients according to the type of treatment received revealed longer survival in patients who had received treatment with surgery + RT + CT (Figure 2).

The five-year survival of IDH-wildtype glioblastoma patients was 3.26%, while the one-year survival after diagnosis was 33.2%.

If we differentiate patients by type of RT received, we found that standard treatment with 60 Gy was the most used and had the highest survival, 18.8 months; survival was less than 10 months with any other treatment scheme.

In terms of the type of CT, patients who received triple therapy (temozolomide + bevacizumab + fotemustine) presented the longest survival. In addition, when stratified for patients treated with or without bevacizumab, a statistically significant increased survival of 20.6 months (95%CI: 13.7–25.1) was observed in patients who received bevacizumab compared with 7.3 months (95%CI: 6.6–8.4) in those who did not (*p* < 0.05).

Regarding sex, the univariate analysis demonstrated that women had a statistically significant worse survival (10 months) than men (13 months) (*p* < 0.05).

### 3.2. IDH-Mutant Astrocytoma Results

#### 3.2.1. Clinical and Demographic Characteristics

The median age at diagnosis of the sample was 56 years (range: 25–88, SD: 14.59). The median age of death was 55.32 (range: 19–80, SD: 18.62). If we stratify IDH-mutant astrocytoma into age groups of 18–30, 30–40, 40–60, and >60 years, we observe that the population of IDH-mutant astrocytoma patients is younger, with patients over 60 years of age representing less than 50% of cases (Table 2).

If we examine IDH-mutant astrocytoma diagnoses per year, to assess whether there has been an increase in incidence in recent years, we observe the following results: 2011: 9, 2012: 13, 2013: 10, 2014: 8, 2015: 14, 2016: 15, 2017: 13, 2018: 16, 2019: 19 and 2020: 20.

**Figure 3:**
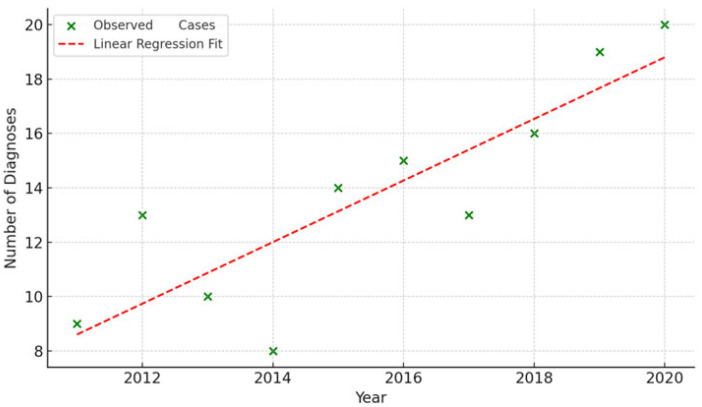
Annual IDH-Mutant Astrocytoma Diagnoses (2011–2020).

A simple linear regression analysis was conducted for IDH-mutant astrocytoma incidence from 2011 to 2020. The model indicated a statistically significant increase in IDH-mutant astrocytoma diagnoses, with an estimated increase of 1.13 cases per year (R^2^ = 0.74, *p* = 0.0015). This suggests a notable rise in IDH-mutant astrocytoma diagnoses during the study period. Figure 3 illustrates the observed data along with the fitted regression line.

In our cohort, there was a slightly higher percentage of men than women (53.5% and 46.5%, respectively). Regarding patients’ functional status measured by the ECOG scale at diagnosis, 55 patients (40.2%) had an ECOG of 0, 60 (43.8%) had an ECOG of 1, and 22 (16%) had an ECOG of 2 or higher.

Regarding the treatment received by patients with IDH-mutant astrocytoma, it is noteworthy that a large majority of patients were treated with surgery + RT + CT (73.72%). The second most common option was biopsy without any adjuvant treatment (Table 3).

Regarding RT treatment, the most used fractionation in our series was 60 Gy (2 Gy fractions), which was administered to 88 patients (77.8%) of the 113 patients who received RT. The second most frequent RT dose option was 54 Gy (2 Gy fractions), administered to 20 patients (17.6%).

#### 3.2.2. Progression-Free Survival (PFS)

**Figure 4:**
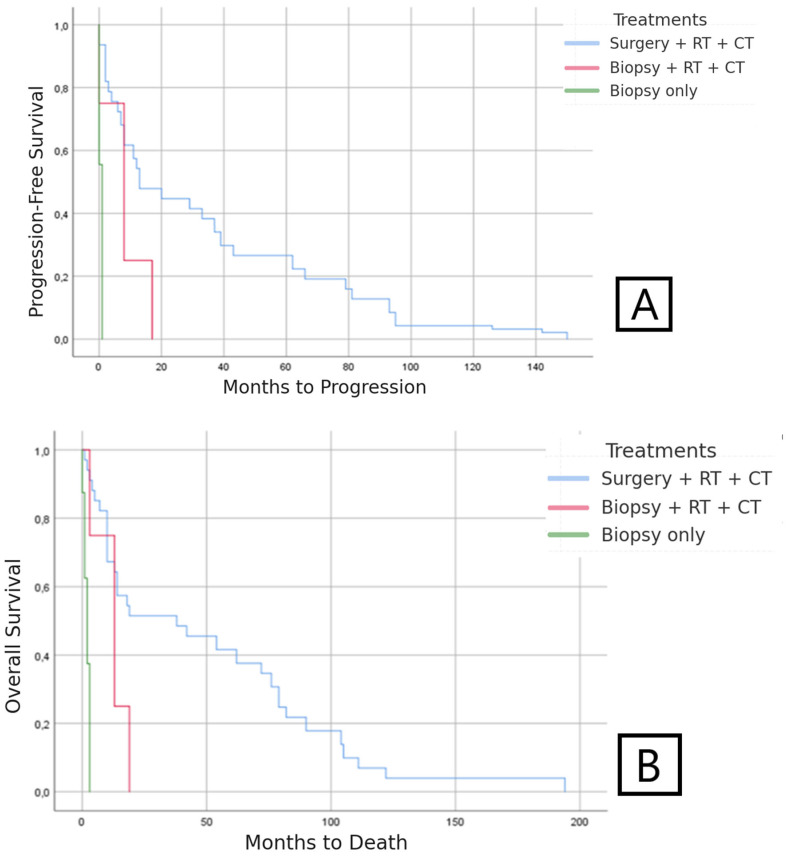
(**A**) PFS according to type of treatment in patients with IDH-mutant astrocytoma. (**B**) OS according to type of treatment in patients with IDH-mutant astrocytoma.

Median PFS was 25 months (95%CI: 20.7–31.9) in the group of IDH-mutant astrocytoma patients. The multivariate analysis showed a significant positive association of PFS with the treatment option of complete surgery + RT + CT compared with patients who did not receive complete surgery, even if the RT + CT treatment was completed. Median time to relapse was 34.25 months (95%CI: 28.6–40.7) in patients who received surgery + RT + CT, and 8.25 months (95%CI: 7.1–8.9) in those patients who could not undergo surgery but did receive RT + CT (Figure 4).

There were significant differences according to sex in this group, where being male afforded a protective factor with a median relapse-free survival of 33.85 months (95%CI: 27.67–41.5) compared with 17.62 months for women (*p* < 0.001).

#### 3.2.3. Overall Survival (OS)

Median OS was 38.4 months (95%CI: 30.7–51.1) in IDH-mutant astrocytoma patients. Prolonged survival was observed in patients treated with surgery + RT + CT, with a median of 50.28 months compared with 12 months in those patients who could not undergo surgery but did receive RT + CT. In patients with biopsy only, the median survival was two months (Figure 4).

Regarding sex, male patients exhibited a statistically significantly higher survival, with a median of 43.21 months (95%CI: 33.4–52.8) compared with 34.63 months (95%CI: 28.7–40.9) in female patients (*p* < 0.001).

The five-year survival of IDH-mutant astrocytoma patients was 29.92%, while the one-year survival was 75.92%.

It is noteworthy that during the study period (2011–2021), no changes were recorded in the therapeutic protocols applied to patients at our center. The majority of cases received surgery followed by radiotherapy and concomitant/adjuvant chemotherapy according to the Stupp protocol, and the introduction of agents such as bevacizumab was limited to the context of disease progression, without relevant modifications over the years.

## 4. Discussion

To date, some studies have evaluated the OS of these tumours; most reported results similar to those in our study, with survival times ranging from 8 to 29 months [18,19,20,21]. Historically, the median OS for IDH-wildtype glioblastoma after the publication of the trial by Stupp et al. was established at 14.6 months [22]. However, the evolution of medicine in recent years, especially in the field of cancer treatment and diagnostic tests for this type of patient, has led to a slight improvement in the results [23,24]. In our sample, a slight improvement in OS was observed, with a median survival of 18.6 months in patients treated with the same regimen as in the Stupp protocol.

On the other hand, OS exhibited a significant difference between the two groups, as in the current literature, where IDH-mutant astrocytoma has greater survival for both PFS and OS (*p* < 0.001). When looking at sex, males had a protective factor in both subgroups. In the IDH-wildtype glioblastoma subgroup, there was no significant difference in PFS between men and women (6.4 and 5.9 months, *p* = 0.458); however, for OS, being of the male gender had a protective factor in our sample, living a median of three months longer (*p* < 0.008).

As for the IDH-mutant astrocytoma group, the sex factor was also important, highlighting that being male was a protective factor since there was a significant difference both at the OS (43.21 months in male patients compared with 34.63 months in female patients) and PFS level (33.85 months for men and 17.62 months for women) (*p* < 0.001). Further studies, with a larger number of patients, are needed to explore this effect in this subset of tumours.

Overall, our data suggest greater OS in fully treated patients, with complete surgery being the feature of greatest benefit in terms of prolonging survival. As a rule, if the patient is in good general condition and there is progression, the cancer treatment is continued as there is increased survival in patients treated with different chemotherapy schemes [25]. Tumours harbouring methylation of the O^6^-methylguanine-DNA-methyltransferase (*MGMT*) gene are known to have a favourable response to temozolomide treatment [26], which could be a potential confounding factor for the differences observed among patients treated with CT.

An important limitation of our study is the absence of data regarding O^6^-methylguanine-DNA-methyltransferase (MGMT) promoter methylation status, which is a well-established prognostic and predictive biomarker in gliomas. The lack of this information may have introduced bias in the interpretation of survival outcomes, particularly in relation to the efficacy of chemotherapy regimens. For example, the apparent overall survival benefit observed in patients treated with bevacizumab or triple therapy (temozolomide + bevacizumab + fotemustine) could, at least in part, reflect the selection of patients with more favorable prognostic features, including potentially MGMT methylation, rather than a direct treatment effect. Therefore, the conclusions regarding chemotherapy efficacy in our cohort must be interpreted with caution.

In our series, patients receiving triple therapy (temozolomide + bevacizumab + fotemustine) showed longer overall survival. However, this observation must be interpreted cautiously, as the retrospective and non-randomized design of the study makes it highly susceptible to selection bias. It is likely that patients who were younger, with better performance status or more favorable prognostic features, were preferentially considered for such intensive regimens. Therefore, no causal relationship between triple therapy and improved survival can be established from our data.

Several studies found an association between survival outcomes and RT treatment, as well as improved clinical outcomes [27,28,29]. The most common RT dose used in our study was 60 Gy, which is the habitual dose in these treatments [22]. Greater survival was found in patients receiving this dose compared with those who received lower doses, which could be related to the patient’s condition or the location of the tumour. In addition, patients who underwent re-irradiation did not have improved survival.

In these patients, the probability of extracranial disease is very low [30,31,32,33]; no disseminated disease was observed in our sample. The vast majority of patients had extension studies; only 15.5% did not have a brain tumour disease extension study, probably because the patients were considered palliative, given the extension of the disease at the brain level and the ill-fated prognosis.

Although the survival observed in our cohort shows a slight improvement compared with historically reported outcomes, we consider that this finding is not related to changes in therapeutic methodology during the study period, since treatment protocols at our center remained stable between 2011 and 2021.

Functional status was better at diagnosis in the IDH-mutant astrocytoma group, probably related to the younger age at diagnosis of this group.

In our cohort, male sex was associated with improved overall survival in both IDH-wildtype glioblastoma and IDH-mutant astrocytoma patients. This finding contrasts with most of the published literature, where either no significant sex-related survival differences are reported, or female sex has been associated with a modest survival advantage [15,16,17,18]. Several possible explanations may account for this unexpected result. First, unrecognized selection or treatment-access biases could have contributed. Second, differences in age at diagnosis between sexes may have influenced survival outcomes, as younger age is a well-established prognostic factor. Finally, biological mechanisms related to hormonal, immunological, or genetic factors, which remain incompletely understood, may also play a role. Further studies with larger sample sizes and external validation are warranted to clarify the nature of these sex-related differences in high-grade gliomas.

When examining the incidence of IDH-wildtype glioblastoma, it appears to be increasing. This finding is in line with several articles published to date [34,35]. The increase in incidence could be related to longer life expectancy due to a reduction in deaths from cardiovascular disease and other causes, although the reasons for this increase in incidence in recent years remain unclear. There is agreement that at least part of this increase is the result of improved diagnostic tests [3,12], although there are differential diagnoses in the initial study [36]. However, improved diagnostic capacity cannot account for all this increase in the incidence of brain tumours. These data, along with evidence suggesting that the incidence may have been growing for many decades, leave open the possibility that environmental exposure may explain some of the rising incidence of brain tumours [3].

Within environmental exposure, ionising radiation is the only firmly established environmental risk factor for brain tumours. Cohort studies of atomic bomb and childhood cancer survivors have shown that cranial radiation is associated with an increased risk of various brain tumours, including meningiomas, gliomas, and nerve sheath tumours. In the case of non-ionising radiation, such as the use of mobile phones, a meta-analysis including data from 22 case-control series established that there was a slightly increased risk associated with mobile phone use; however, there were potential confounders [37]. Another conclusion of this meta-analysis was that the risk appeared after an induction period of 10 years or longer. Due to inconsistencies observed in studies and potential biases in case-control studies, in 2011, the WHO/IARC classified radiofrequency electromagnetic fields as possibly carcinogenic to humans (Group 2B, i.e., a causal association is considered credible, but when chance, bias, or confounding cannot be ruled out with reasonable confidence).

In addition to ionising and non-ionising radiation, other environmental exposures have been postulated as potential contributors to the increasing incidence of glioblastoma [38]. Experimental studies in rodents have demonstrated that alkylating agents, particularly N-nitrosamines, are capable of inducing brain tumours. These compounds are present in processed foods, tobacco smoke, contaminated water, and certain industrial environments. Moreover, air pollution, which is frequently associated with increased exposure to nitrosamine precursors and oxidative stress, has also been suggested as a potential environmental risk factor for gliomas [39,40]. While definitive causal links in humans remain to be established, these factors warrant further investigation given the consistent epidemiological trends.

In addition, new causative agents such as radon have been observed in recent years. In the United States, a study was carried out suggesting a higher incidence of non-malignant brain tumours in regions with high exposure to particles and radon. These findings provide insight into the unexplained variation in tumour incidence, although future studies are needed to validate these findings in other populations [41].

## 5. Limitations and Future Directions

This study has inherent limitations due to its retrospective design and the relatively small number of patients within the IDH-mutant astrocytoma subgroup. Additionally, the accuracy and completeness of electronic medical records may vary, introducing potential information bias. A further limitation concerns selection bias, as part of the data collection was performed during a period in which the electronic medical record system was not yet fully implemented across the institution. Consequently, some relevant clinical data may have been underreported or inconsistently documented.

One of the main limitations of this study is that all IDH-mutant astrocytic tumors were analyzed together as a single group, regardless of WHO grade (2, 3, or 4). This introduces biological and clinical heterogeneity, as prognosis and treatment strategies differ substantially across grades. Unfortunately, stratification by grade was not possible due to incomplete data in the historical cohort. Therefore, the reported survival outcomes for IDH-mutant astrocytoma should be interpreted as an average across a mixed-prognosis population, and they cannot be generalized to any specific WHO grade.

Although this classification reflects current WHO CNS guidelines, it inherently introduces biological and clinical heterogeneity, as prognosis and treatment strategies differ substantially between lower-grade and higher-grade astrocytomas. The decision to group these tumors was made to preserve statistical power given the sample size, but it should be considered when interpreting survival outcomes and treatment-related comparisons within this subgroup.

Conversely, a notable strength of this study lies in the relatively large number of patients diagnosed with IDH-wildtype glioblastoma, enabling meaningful survival and epidemiological analysis in a real-world clinical setting. Furthermore, this article may help us gain a better understanding of the incidence of this type of tumour and help pilot studies on organ donation in cancer patients with malignant primary brain tumours, such as that being developed at the Virgen de la Arrixaca University Clinical Hospital [42], as well as the extensive indications for donation [43].

## 6. Conclusions

In our experience, we have observed the aggressive potential of IDH-wildtype glioblastoma, with very low survival rates (33% one-year survival), and a progressive increase in incidence in recent years. Our results suggest that surgery in combination with radiotherapy and chemotherapy is associated with improved overall survival, although, given the retrospective nature of the study and the absence of key prognostic data such as MGMT promoter methylation status, these findings should be interpreted with caution. In terms of sex, male patients showed longer survival, with a more marked difference observed in patients with IDH-mutant astrocytoma. This group, who were younger, had a better survival, with an average of almost three years.

## Figures and Tables

**Table 1:** Percentage of patients by age range.

Percentage	IDH-Mutant Astrocytoma Age (Years)	Percentage
18–30	18–30	6.5
30–40	30–40	14.7
40–60	40–60	34.7
>60	>60	44.1

**Table 2:** Type of treatment received by patients with IDH-wildtype glioblastoma and survival outcomes by type of treatment.

	Number of Patients	Percentage	Progression-Free Survival (Months)		Overall Survival (Months)	
Surgery + CT + RT	309	58.30%	9.493		18.645	
Surgery + RT	28	5.28%	4.742		7.871	
Biopsy only	18	3.40%	0.775	*p* < 0.001	2.523	*p* < 0.001
Biopsy + RT + CT	115	21.70%	5.059		6.882	
Biopsy + CT	60	11.32%	0.698		2.651	

**Table 3:** Type of treatment received by patients with IDH-mutant astrocytoma and survival results by type of treatment.

	Total N	Percentage	Progression-Free Survival (Months)		Overall Survival (Months)	
Surgery + RT + CT	101	73.72	34.255		50.287	
Biopsy + RT + CT	12	8.76	8.250	*p* < 0.001	12.000	*p* < 0.001
Biopsy only	24	17.52	0.556		1.875	
Overall	137	100	25.068		38.453	

## Data Availability

The original contributions presented in this study are included in the article/Supplementary Material. Further inquiries can be directed to the corresponding author.

## Supplementary Materials

The following supporting information can be downloaded at: https://www.mdpi.com/article/10.3390/medsci13040233/s1. Table S1: Summary of the pathological diagnoses of the included brain tumor cases.
