# Supplementary material for: Incidence and Survival of IDH-Wildtype Glioblastoma and IDH-Mutant Astrocytoma by Treatment and Sex: A Regional Study in Spain (2011–2021)
Source: Med Sci (Basel). 2025 Oct 14;13(4):233. doi: 10.3390/medsci13040233 (PMC12551061; doi:10.3390/medsci13040233)
Supplement: Supplementary file 1 [file medsci-13-00233-s001.zip › medsci-3814446-supplementary.pdf]

**Diagnosis.**	**Number of patients**	**Percentage**
Glioblastoma, IDH-wildtype	530	50.14
Astrocytoma, IDH-mutant	137	12.96
Meningiomas	122	11.54
Grade II astrocytoma	67	6.34
Ependymoma	55	5.20
Anaplastic oligodendroglioma	39	3.69
Anaplastic ependymoma	26	2.46
Gliosarcoma	18	1.70
Grade II oligodendroglioma	14	1.32
Trunk glioma	12	1.14
Medulloblastoma	9	0.85
Glomus jugulare	6	0.57
Pilomyxoid astrocytoma	5	0.47
Gemistocytic astrocytoma	4	0.38
Meningothelial meningioma	3	0.28
Giant cell astrocytoma	2	0.19
Esthesioneuroblastoma	2	0.19
leomorphic xanthoastrocytoma	2	0.19
Grade II pineal tumour	2	0.19
de IV primitive neuro-ectodermal tumour (PNETs)	2	0.19
Total	1057	100.0

Table 1. Diagnoses of the patients in the study.
